# Clinicopathological Factors Affecting Survival and Recurrence after Initial Hepatectomy in Non-B Non-C Hepatocellular Carcinoma Patients with Comparison to Hepatitis B or C Virus

**DOI:** 10.1155/2014/975380

**Published:** 2014-03-13

**Authors:** Yoshihiro Okuda, Shugo Mizuno, Taizou Shiraishi, Yasuhiro Murata, Akihiro Tanemura, Yoshinori Azumi, Naohisa Kuriyama, Masashi Kishiwada, Masanobu Usui, Hiroyuki Sakurai, Masami Tabata, Tomomi Yamada, Shuji Isaji

**Affiliations:** ^1^Department of Hepatobiliary-Pancreatic and Transplant Surgery, Mie University School of Medicine, 2-174 Edobashi, Tsu, Mie 514-0001, Japan; ^2^Department of Oncologic Pathology, Mie University Graduate School of Medicine, Tsu, Mie 514-0001, Japan; ^3^Department of Translational Medical Science, Mie University School of Medicine, 2-174 Edobashi, Tsu, Mie 514-0001, Japan

## Abstract

We evaluated clinicopathological factors affecting survival and recurrence after initial hepatectomy in non-B non-C (NBNC) hepatocellular carcinoma (HCC) patients with comparison to hepatitis B or C virus, paying attention to relationship between alcohol consumption and histopathological findings. The medical records on the 201HCC patients who underwent initial hepatectomy between January 2000 and April 2013 were retrospectively reviewed. NBNC patients had higher prevalence of hypertension (47.4%), diabetes mellitus (35.5%), alcohol consumption (>20 g/day) (61.8%), and preserved liver function than hepatitis B or C patients. The 5-year survival rate of NBNC patients (74.1%) was significantly better than hepatitis B (49.1%) or C (65.0%) patients (NBNC versus B, *P* = 0.031). Among the NBNC patients, there was no relationship between alcohol consumption and clinicopathological findings including nonalcoholic fatty liver disease activity score (NAS). However, the 5-year OS and RFS rates in the alcohol-unrelated NBNC patients tend to be better than in the alcohol-related. By multivariate analysis, independent factors for OS in NBNC patients were Child-Pugh B/C, intrahepatic metastasis (im), and extrahepatic recurrence. NBNC patients, who were highly associated with lifestyle-related disease and preserved liver function, had significantly better prognosis compared to hepatitis B/C patients; however, there was no association between alcohol consumption and histopathological findings.

## 1. Introduction

Primary liver cancer including hepatocellular carcinoma (HCC) is the fifth most frequently diagnosed cancer worldwide [1], and chronic viral hepatitis and liver cirrhosis following hepatitis B virus (HBV) or hepatitis C virus (HCV) infection had been reported to be responsible for most HCCs [2]. Although Japan has had one of the highest incidence rates of HCC associated with chronic HCV infection [3], a nationwide follow-up survey by the Liver Cancer Study Group of Japan found that the proportion of hepatitis virus-related HCC had decreased over the previous decade, possibly due to the promotion of antiviral therapy, whereas the number of other HCC patients (mostly non-B non-C HCC: NBNC-HCC) had more than doubled during the same period from 6.8% to 17.3% [4].

It remains controversial whether NBNC-HCC patients have comparable prognosis to HCC patients with HBV or HCV. In the previous studies, NBNC-HCC patients had a poorer prognosis than hepatitis virus-related HCC patients because NBNC-HCCs were often detected at an advanced stage incidentally without followup [5–7]. In contrast, a few studies reported that the postoperative outcome of NBNC patients were better than that of HBV or HCV patients, because hepatitis virus-related patients had poor liver function, more advanced tumors, and multicentric carcinogenesis in the remnant liver [8, 9]. These conflicting results are considered to be due to the fact that the clinicopathological characteristics in NBNC patients still remain unclear because various clinical factors including age, gender, alcohol consumption, and DM are involved in the carcinogenesis and progression of NBNC-HCCs.

Nonalcoholic fatty liver disease (NAFLD) and nonalcoholic steatohepatitis (NASH) have recently assumed increasing attention for their relationship with HCC [10–15]. Although laboratory test and radiographic findings may be suggestive of NAFLD, histological evaluation is still the gold standard for accurate diagnosis of NAFLD/NASH by assessing the degree of steatosis, the distinct necroinflammatory lesions and fibrosis of NASH, and distinguishing NASH from simple steatosis or steatosis with inflammation. Recently, feature-based semiquantitative scoring system of NAFLD, NAFLD activity score (NAS), was developed by the pathology committee of the NASH Clinical Research Network [16], and the feasibility of this score was reported as optimal scoring system for predicting steatohepatitis [17]. Nevertheless, there have been few reports examining the prevalence of steatohepatitis in NBNC-HCC patients and/or regarding the relationship between their surgical outcomes and degree of steatohepatitis.

The aim of the present study was to clarify the clinicopathological features of the NBNC-HCC patients who underwent initial hepatectomy by evaluating the factor affecting survival and recurrence after hepatectomy, paying attention to relationship between alcohol consumption and histopathological findings including NAS.

## 2. Patients and Methods

### 2.1. Patient Groups

#### 2.1.1. Comparison among Three Groups according to Hepatitis Virus

We retrospectively reviewed a total of 201 primary HCC patients who consecutively underwent initial hepatectomy at the Mie University Hospital between January 2000 and April 2013. All patients were divided into the following three groups based on the presence of serum antigens/antibodies for hepatitis virus B/C: group B (*n* = 32) which were positive for HBs-Ag and negative for HCV-Ab; group C (*n* = 93) which were negative for HBs-Ag and positive for HCV-Ab; and group NBNC (*n* = 76) which were negative for both HBs-Ag and HCV-Ab. In the present study, none of NBNC-HCC patients included the known etiologies such as primary biliary cirrhosis, other biliary cirrhosis (such as primary sclerosing cholangitis and secondary biliary cirrhosis), autoimmune hepatitis, metabolic diseases (Wilson's disease, hemochromatosis, and glycogen storage disease), congestive diseases including Budd-Chiari syndrome, and parasitic diseases.

#### 2.1.2. Subgroup Analysis of NBNC-HCC according to Alcohol Consumption

According to the summary of talks presented at the American Association for the Study of Liver Diseases Clinical Single Topic Conference on NASH [18],* a reasonable cut-off level of daily alcohol consumption, which would be the threshold at which steatohepatitis becomes alcohol-related, is 20 g* ethanol. Therefore, all patients in group NBNC (*n* = 76) were further divided into the following two groups based on the daily alcohol consumption: NALP (nonalcoholic patients) (*n* = 30) in which alcohol consumption was less than 20 g ethanol/day and ALP (alcoholic patients) (*n* = 46) in which it was 20 g or more ethanol/day. A history of alcohol consumption was obtained from interviews with patients or their families.

### 2.2. Methods

We compared various factors in the three groups according to hepatitis virus in all patients and alcohol consumption in group NBNC in order to clarify clinicopathological characteristics of each group, including (1) lifestyle-related factors such as obesity, hypertension (HTN), and DM; (2) preoperative clinical factors such as neutrophil-to-lymphocyte ratio (NLR), indocyanine green retention rate at 15 minutes (ICG R15), Child-Pugh class (A or B/C), tumor size (maximum diameter and more than 10 cm or less) on dynamic CT scan, Barcelona Clinic Liver Cancer (BCLC) stage (0/A or B/C) [19], and Milan criteria (within or beyond) [20]; (3) surgical factors such as surgical procedures (more than 2 sectionectomy or less) and surgical curability (R0 or R1/2); (4) pathological factors of the resected specimen such as intrahepatic metastasis (im) and histological findings of noncancerous liver; and (5) postoperative factors such as Clavien-Dindo classification for postoperative complications (I/II or III–V) [21] and posthepatectomy liver failure (PHLF) grade (0/A or B/C) [22].

### 2.3. Definition of Obesity, Hypertension, DM, and Cigarette Smoking

Obesity was defined as body mass index (BMI) ≧ 25 kg/m^2^ according to the criteria of the Japan Society for the Study of Obesity [23]. Hypertension was diagnosed by a systolic blood pressure of more than 140 mmHg and/or diastolic blood pressure of more than 90 mmHg or by prescription of antihypertensive agents. DM was diagnosed according to the 2006 World Health Organization (WHO) criteria [24]. Cigarette smoking includes past history of smoking.

### 2.4. Definition of NLR

The NLR was calculated from the differential count by dividing the absolute neutrophil count by the absolute lymphocyte count. The cut-off level of NLR was defined as 4.0 according to the previous report [25].

### 2.5. Determination of the Type of Hepatectomy

After diagnosis of HCC, the most appropriate surgical procedure was selected based on the tumor characteristics and underlying the hepatic functional reserve of each patient by using ICGR15, LHL15, and hyaluronic acid according to our previous report [26].

### 2.6. Patient Follow-Up after Hepatectomy

Followup after surgery comprised periodic blood tests and monitoring of the tumor markers (serum *α*-fetoprotein (AFP) level and des-r-carboxyprothrombin (DCP) level). Dynamic CT images and/or MRI of the liver were carried out every 3-4 months until two years after hepatectomy and thereafter they were performed every 6 months. Chest CT, whole abdominal CT, brain MRI, and bone scintigraphy were done if recurrence of extrahepatic HCC was suspected.

### 2.7. Histological Examination of Noncancerous Liver in Group NBNC

In group NBNC, the noncancerous regions of the surgical specimens were stained with hematoxylin and eosin (H&E) and Masson's trichrome and reevaluated by a single experienced pathologist (T.S.) who was unaware of the laboratory data and the clinical course, according to the National Institutes of Health-sponsored NASH Clinical Research Network system, which is called the Kleiner's scoring system [24] as follows. The elements of NAS and the stage of fibrosis were scored with separate scores for steatosis (0–3), hepatocellular ballooning (0–2), lobular inflammation (0–3), and fibrosis (0–4). NAS was the sum of the first three features and ranged from 0 to 8. Fibrosis according to the NAS was scored from 0 to 4 (0: none, 1: perisinusoidal or periportal, 2: perisinusoidal and periportal, and 3: bridging and 4: cirrhosis). NAS of 5 or more correlated with a diagnosis of NASH, and NAS of less than 3 were diagnosed as “not NASH.”As the authors emphasized, the NAS was originally designed to assess overall histologic change before and after therapeutic intervention trials and was not intended as numeric values to replace a pathologist's diagnostic determination of steatohepatitis.

### 2.8. Statistical Analysis

All continuous values except for patient age are presented as medians and ranges (minimum–maximum). Patient age is presented as mean ± SD. Continuous variables were compared using Student's *t*-test or one-way analysis of variance (ANOVA) and Mann-Whitney* U*-test or Kruskal-Wallis method, and categorical variables were compared using Pearson's chi-squared test. The overall survival (OS) and recurrence-free survival (RFS) were calculated using the Kaplan-Meier method and tested using the log-rank test. In evaluating factors affecting OS, the Cox regression model with stepwise variable selection was used for multivariate analysis. Statistical data analysis was performed using the SPSS program, version 20.0 (SPSS, Chicago, Ill. USA). A *P* value less than 0.05 was considered statistically significant.

## 3. Result

### 3.1. Comparison among Three Groups according to Hepatitis Virus

#### 3.1.1. Preoperative Findings

The lifestyle-related and preoperative clinical findings in the three groups are listed in Table 1. The mean age of patients was the highest in group NBNC followed by groups C and B (*P* < 0.001). The patients in group NBNC had significantly higher prevalence of hypertension (47.4%) and DM (35.5%) than those in the other groups (*P* = 0.004 and *P* = 0.041, resp.). The incidence of patients with alcohol consumption of 20 g/day or more was the highest in group NBNC followed by groups C and B. In the laboratory data, the platelet counts and PT were the highest in group NBNC followed by groups C and B (*P* = 0.020 and *P* = 0.002, resp.). ICG R15 was the lowest in group NBNC followed by groups B and C (*P* = 0.027). AFP levels were the lowest in group NBNC (*P* < 0.001) followed by groups C and B. The percentage of the patients with tumors more than 10 cm in diameter was higher in groups NBNC (18.4%) and B (21.9%) than in group C (2.2%) (*P* = 0.001 and *P* = 0.001, resp.). The proportion of the patients with tumors beyond Milan criteria was higher in groups NBNC (50.0%) and B (46.9%) than in group C (28.0%) (*P* = 0.009).

#### 3.1.2. Intraoperative, Pathological, and Postoperative Factors

As shown in Table 2, the proportion of the patients who needed more than two sectionectomy was significantly higher in groups NBNC and B than in group C (*P* = 0.002). There were no significant differences in operative times, intraoperative blood loss, and surgical curability. In pathological examination, the proportion of simple nodular type was significantly lower in groups NBNC and B than in group C (*P* = 0.025), and the proportion of vp(+) was the second highest in group NBNC (*P* = 0.016). The proportion of NL in noncancerous liver was significantly higher in group NBNC than groups B and C and the proportion of LC was the lowest in group NBNC followed by groups B and C (*P* < 0.001 and *P* = 0.020, resp.).

#### 3.1.3. Cumulative Overall Survival and Recurrence-Free Survival

**Figure 1:**
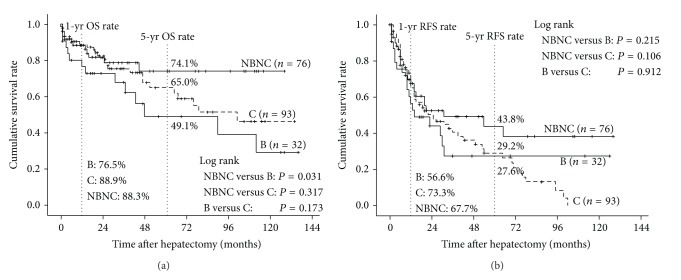
Comparisons of overall survival and recurrence-free survival rates after hepatectomy between patients in groups B, C, and NBNC. (a) Overall survival. The survival rate of NBNC patients (unbroken thick line) was significantly better than that of B patients (unbroken line, *P* = 0.031). (b) Recurrence-free survival. There were no significant differences in survival rates of three groups.

The survival curves of OS and RFS rates are shown in Figure 1. The median follow-up periods were 20 months (range, 0–136 months) for all patients. The 1- and 5-year OS rates were 88.3% and 74.1% in group NBNC, 88.9% and 65.0% in group C and 76.5%, and 49.1% in group B, respectively, showing significant difference between groups NBNC and B (*P* = 0.031). The 1- and 5-year RFS rates were 67.6% and 43.8% in group NBNC, 73.3% and 29.2% in group C, and 56.6% and 27.6% in group B, respectively. There was no significant difference in RFS rates between the three groups.

### 3.2. Subgroup Analysis of NBNC-HCC according to Alcohol Consumption

The lifestyle-related and preoperative clinical findings in the two subgroups are listed in Table 3. In NALP, the proportions of female and noncigarette smokers were significantly higher than those in ALP (*P* = 0.001 and *P* = 0.007, resp.). As shown in Table 4, there were no significant differences in intraoperative, pathological, and postoperative factors between the two subgroups.

Table 5 shows the data based on the scores of steatosis, lobular inflammation, ballooning and fibrosis, the NAS, and NAS with fibrosis score. Moderate and severe steatosis, which is score of 2 (33–66%) and 3 (66% or more), was found only in 2 patients (6.6%) in NALP and 2 patients (4.3%) in ALP. Furthermore, NASH, which was defined as NAS of 5 or more, was found in 2 patients (6.7%) in NALP. There were no significant differences in all of the histopathological factors, NAS and NAS with fibrosis score between the two subgroups.

**Figure 2:**
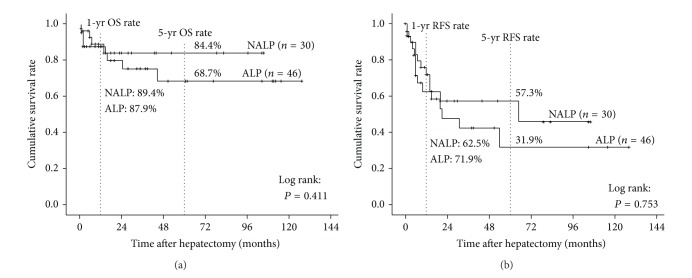
Comparisons of overall survival and recurrence-free survival rates after hepatectomy between patients in group NBNC. (a) Overall survival. There were no significant differences in survival rates of two subgroups. (b) Recurrence-free survival. There were no significant differences in survival rates of two subgroups.

The survival curves of OS and RFS in NALP and ALP are shown in Figure 2. The median follow-up periods were 15 months (range, 0–128 months). The 1- and 5-year OS rates in NALP versus ALP were 89.4% versus 87.9% and 84.4% versus 68.7%, respectively, showing no significant difference. The 1- and 5-year RFS rates in NALP versus ALP were 62.5% versus 71.9% and 57.3% versus 31.9%, respectively, showing no significant difference.

Table 6 shows the results obtained by multivariate analysis of factors influencing on OS. Child-Pugh B/C, im(+) and extrahepatic recurrence were identified as independent indicators of OS.

## 4. Discussion

To clarify the clinicopathological features of the NBNC-HCC patients with initial hepatectomy, we first compared the patient- and tumor-related factors among the three groups B, C, and NBNC. In the patient-related factors, NBNC patients were characterized by association with lifestyle-related diseases (DM, HTN, and alcohol consumption) and preserved liver function (platelet counts, PT, ICG R15, and higher prevalence of NL in noncancerous liver).

To the best of our knowledge, there have been seven reports comparing clinicopathological features among the patients with hepatitis virus-related and nonhepatitis virus-related HCCs all of which were from Japan after 2000, and, among them, four included less than 30 NBNC patients [8, 9, 27, 28] and the other three after 2012 included more than 60 NBNC patients (*n* = 60, 129, and 168, resp., [6, 29, 30]). Therefore, the results of our study were compared with those of the latter three reports. However, none of them focused on lifestyle-related diseases precisely, especially in NALFD/NASH. All of the previous three reports demonstrated that NBNC patients had much better preoperative liver function (platelet count and ICG R15), and Kudo et al. [30] reported that the incidence of liver cirrhosis was significantly lower in NBNC patient (34%) than in HBV (52%) and HCV (56%) patients, which were similar to our results.

As of the tumor-related factors, Kaibori et al. [6] reported that NBNC and HBV patients had significantly higher AFP and DCP levels than HCV patients, while the other two reports showed no difference in AFP and DCP levels among the three groups. The two reports demonstrated that tumor sizes were larger in NBNC (median [cm]: 5.57, 5.8) and HBV (median [cm]: 5.43, 5.4) patients than in HCV (median [cm]: 3.55, 4.0) patients, which were similar to our results. All of the three reports showed no significant differences in microvascular invasion and surgical margin among the three groups, which were similar to our results.

As of intraoperative and postoperative factors, the proportion of the patients with two sectionectomy was significantly higher in groups NBNC and B than in group C, reflecting tumor size in each group. There were no significant differences in operative times, intraoperative blood loss postoperative morbidity, and 90-day mortality among the three groups.

In the present study, the 5-year OS rates were 74.1% in group NBNC, 65.0% in group C, and 49.1% in group B, showing significant difference between groups NBNC and B, while there was no significant difference in RFS rates between the three groups. The previous three reports [6, 29, 30], however, demonstrated conflicting results: two reports showed significantly better survival in NBNC patients than in HBV and HCV patients, while the other report showed no survival difference in the three groups. In the former two reports [6, 30], since tumor-related factors did not differ significantly among the three groups, it was considered that good liver function at the initial hepatectomy might prevent early recurrence in NBNC patients. In the latter report [29], they suggested that the lack of difference in survival after curative resection might be because NBNC patients were associated with larger tumors but a better hepatic functional reserve. In our study, as of patient-related factors in NBNC patients, hepatic functional reserve was best preserved and the proportion of NL in noncancerous liver was the highest. While, as of tumor-related factors in NBNC patients, although AFP levels were the lowest, the percentages of the patients with tumors more than 10 cm in diameter and tumors beyond Milan criteria were high, showing similar percentages to HBV patients. Furthermore, the proportion of the patients who needed more than two sectionectomy was higher in groups NBNC and B than in group C. Taking these results together, it was considered that well-preserved liver function after initial hepatectomy in NBNC patients might contribute to survival benefit even if tumor recurrence occurred.

We furthermore performed subgroup analysis of NBNC patients based on alcohol consumption using cut-off level of ethanol 20 g/day. The proportions of female and noncigarette smokers were significantly higher in NALP than in ALP. Between the two subgroups, there were no significant differences in all of the other clinicopathological factors, including preoperative liver function and NAS. The OS and RFS rates in NALP were higher than those in ALP, although there were no statistical significant differences. This might be because the patient number was not so large and further investigation will be needed to clarify the influence of alcohol consumption on their prognosis. Kudo et al. [30] also analyzed the 163 NBNC patients by dividing the three groups according to alcohol consumption: nonalcoholic liver disease (non-ALD: ethanol less than 20 g/day), mild ALD (ethanol 20 g–80 g/day), and severe ALD (ethanol 80 g/day or more). They revealed that severe ALD was associated with being male, small tumor size, and LC, although they did not examine the degree of steatohepatitis. They concluded that preoperative excessive alcohol intake decreased DFS rate of HCC occurrence after surgery.

In multivariate analysis of factors influencing on OS in group NBNC, Child-Pugh B/C, im(+), and extrahepatic recurrence were identified as independent indicators of OS. Several previous studies on prognostic factors in NBNC-HCC patients identified gender, serum albumin level, DCP, tumor size, tumor capsule, and tumor differentiation as significant independent factors for OS [6, 9, 29–31]. Therefore, in terms of OS, preoperative liver functions such as serum albumin and Child-Pugh class are important as patient-related factor and degree of tumor malignancy as tumor-related factor, showing the results similar to those in hepatitis virus-related HCC patients.

In conclusion, NBNC-HCC patients, who were characterized by association with lifestyle-related disease and preserved liver function, had significantly better prognosis compared to HBV and HCV patients. Significant prognostic factors in NBNC patients were preoperative poor hepatic functional reserve and tumor extension, which were similar to those in hepatitis virus-related HCC patients. In an attempt to clarify the association of NALFD/NASH with HCC by examining NAS in the resected specimens, however, we could not demonstrate any association.

## Figures and Tables

**Table 1:** Lifestyle-related and preoperative clinical factors.

	B (*n* = 32)	C (*n* = 93)	NBNC (*n* = 76)	*P* value
Age (years)	61.1 ± 13.6	67.2 ± 6.6	71.0 ± 9.2	**<0.001**
Gender (male/female)	23/9	70/23	67/9	0.058
Obesity	3 (9.4%)	17 (18.3%)	15 (19.7%)	0.412
Hypertension	6 (18.8%)	26 (28.0%)	36 (47.4%)	**0.004**
DM	5 (12.0%)	20 (21.5%)	27 (35.5%)	**0.041**
Cigarette smoking	19 (59.4%)	55 (59.1%)	47 (61.8%)	0.933
Ethanol ≧ 20 g	5 (15.6%)	38 (40.9%)	46 (60.5%)	**<0.001**
Albumin (g/dL)	3.6 (2.3–4.5)	3.7 (2.8–4.5)	3.8 (2.5–5.0)	0.104
Total bilirubin (mg/dL)	0.5 (0.2–1.2)	0.5 (0.2–1.4)	0.5 (0.2–1.3)	0.873
Platelet count (×10^4^/mm^3^)	14.0 (4.3–48.9)	16.1 (4.4–51.0)	19.2 (6.7–51.6)	**0.020**
NLR	2.21 (0.79–4.90)	1.92 (0.52–14.16)	2.16 (0.67–12.80)	0.271
PT (%)	82.9 (64.3–105.0)	90.0 (63.4–122.7)	95.4 (54.8–131.4)	**0.002**
ICG R15 (%)	12.7 (4.4–19.7)	14.0 (2.8–42.3)	10.9 (1.6–36.7)	**0.027**
Child-Pugh B/C	2 (6.3%)	5 (5.4%)	6 (7.9%)	0.802
AFP (ng/mL)	110.0 (1–605100)	14.0 (2–11675)	6.0 (1–48157)	**<0.001**
DCP (mAU/mL)	267.0 (10–174400)	148.0 (10–23950)	87.0 (11–85330)	0.653
Multiple tumors	10 (31.3%)	25 (26.9%)	22 (28.9%)	0.885
Tumor size (cm)	5.2 (1.0–20.0)	3.0 (0.7–11.0)	4.5 (1.0–17.0)	**0.001**
≧10 cm	7 (21.9%)	2 (2.2%)	14 (18.4%)	**0.001**
BCLC stage B/C	8 (25.0%)	16 (17.2%)	18 (23.7%)	0.484
Beyond Milan criteria	15 (46.9%)	26 (28.0%)	38 (50.0%)	**0.009**
Preoperative TACE	17 (53.1%)	60 (64.5%)	42 (55.3%)	0.356

DM: diabetes mellitus; NLR: neutrophil-to-lymphocyte ratio; PT: prothrombin time; ICG R15: indocyanine green retention rate at 15 minutes; AFP: serum *α*-fetoprotein; DCP: des-r-carboxyprothrombin; BCLC: Barcelona Clinic Liver Cancer; TACE: transcatheter arterial chemoembolization.

**Table 2:** Intraoperative, pathological and postoperative factors.

	B (*n* = 32)	C (*n* = 93)	NBNC (*n* = 76)	*P* value
≧2 sentionectomy	13 (40.6%)	17 (18.3%)	32 (42.1%)	**0.002**
Operative Time (min.)	352 (177–658)	333 (135–750)	367 (173–983)	0.142
Blood loss (g)	1137 (38–8488)	1182 (5–8307)	1226 (200–36000)	0.590
Curability R0	27 (84.4%)	88 (94.6%)	70 (92.1%)	0.181
Simple nodular type	12 (37.5%)	54 (58.1%)	30 (39.5%)	**0.025**
Poorly differentiated	6 (18.8%)	14 (15.1%)	6 (7.9%)	0.218
fc(+)	24 (75.0%)	63 (67.7%)	44 (57.9%)	0.182
vp(+)	17 (53.1%)	24 (25.8%)	28 (36.8%)	**0.016**
vv(+)	5 (15.6%)	4 (4.3%)	7 (9.2%)	0.109
im(+)	6 (18.8%)	7 (7.5%)	7 (9.2%)	0.181
Non-cancerous liver NL	1 (3.1%)	1 (1.1%)	23 (30.3%)	**<0.001**
CH	17 (53.1%)	44 (47.3%)	29 (38.2%)	0.288
LC	14 (43.8%)	48 (51.6%)	23 (30.3%)	**0.020**
Clavien-Dindo ≧ III	5 (15.6%)	19 (20.4%)	12 (15.8%)	0.688
PHLF B/C	7 (21.9%)	28 (30.1%)	18 (23.7%)	0.526
90-day mortality	4 (12.5%)	6 (6.5%)	6 (7.9%)	0.552
Intrahepatic recurrence	13 (40.6%)	47 (50.5%)	25 (32.9%)	0.068
Extrahepatic recurrence	7 (21.9%)	8 (8.6%)	7 (9.2%)	0.096

fc: formation of capsule; vp: microscopic portal vein invasion; vv: microscopic hepatic vein invasion; im: intrahepatic metastasis; NL: normal liver; CH: chronic hepatitis; LC: liver cirrhosis; PHLF: posthepatectomy liver failure.

**Table 3:** Lifestyle-related and preoperative clinical factors in Group NBNC according to alcohol consumption.

	NALP (*n* = 30)	ALP (*n* = 46)	*P* value
Age (years)	73.3 ± 6.3	69.7 ± 10.9	0.491
Gender (Male/Female)	22/8	45/1	**0.001**
Obesity	7 (23.3%)	8 (17.4%)	0.525
Hypertension	13 (43.3%)	23 (50.0%)	0.569
DM	11 (36.7%)	16 (34.8%)	0.867
Cigarette smoking	13 (43.3%)	34 (73.9%)	**0.007**
Albumin (g/dL)	3.7 (2.5–5.0)	3.9 (2.8–4.6)	0.658
Total Bilirubin (mg/dL)	0.5 (0.2–1.3)	0.5 (0.2–0.9)	0.834
Platelet count (×10^4^/mm^3^)	20.8 (8.0–40.4)	17.8 (6.7–51.6)	0.100
NLR	2.12 (0.87–12.80)	2.15 (0.79–7.43)	0.461
PT (%)	93.0 (54.8–131.4)	100.5 (63.4–131.4)	0.714
ICG R15 (%)	11.4 (0.3–29.0)	9.9 (1.6–31.5)	0.124
AFP (ng/mL)	7.0 (1–48157)	5.0 (1–7153)	0.398
DCP (mAU/mL)	94.0 (14–85330)	80.0 (11–15387)	0.741
Child-Pugh B/C	3 (10.0%)	3 (6.5%)	0.583
Multiple tumors	10 (33.3%)	12 (26.1%)	0.496
Tumor size (cm)	5.0 (1.0–15.0)	3.8 (1.2–17.0)	0.229
≧10 cm	6 (20.0%)	8 (17.4%)	0.774
BCLC stage B/C	9 (30.0%)	9 (19.6%)	0.296
Beyond Milan criteria	19 (63.3%)	19 (41.3%)	0.060
Preoperative TACE	15 (50.0%)	27 (58.7%)	0.456

NALP: non-alcoholic patients; ALP: alcoholic patients; DM: diabetes mellitus; NLR: neutrophil-to-lymphocyte ratio; PT: prothrombin time; ICG R15: indocyanine green retention rate at 15 minutes; AFP: serum *α*-fetoprotein; DCP: des-r-carboxyprothrombin; BCLC: Barcelona Clinic Liver Cancer; TACE: transcatheter arterial chemoembolization.

**Table 4:** Intraoperative, pathological and postoperative factors in Group NBNC according to alcohol consumption.

	NALP (*n* = 30)	ALP (*n* = 46)	*P* value
≧2 sentionectomy	13 (43.3%)	19 (41.3%)	0.861
Operative Time (min.)	385 (173–983)	340 (202–572)	0.073
Blood loss (g)	1182 (200–36000)	1280 (200–33478)	0.663
Curability R0	29 (96.7%)	41 (89.1%)	0.449
Simple nodular type	9 (30.0%)	21 (45.7%)	0.172
Poorly differentiated	2 (6.7%)	4 (8.7%)	0.748
fc(+)	18 (60.0%)	26 (56.5%)	0.764
vp(+)	12 (40.0%)	16 (34.8%)	0.645
vv(+)	2 (6.7%)	5 (10.9%)	0.536
im(+)	2 (6.7%)	5 (10.9%)	0.536
Non-cancerous liver NL	8 (26.7%)	15 (32.6%)	0.582
CH	12 (40.0%)	17 (37.0% )	0.789
LC	9 (30.0% )	14 (30.4%)	0.968
Clavien-Dindo ≧ III	7 (23.3%)	5 (10.9%)	0.145
PHLF B/C	9 (30.0%)	9 (19.6%)	0.296
90-day mortality	1 (3.0%)	5 (10.9%)	0.234
Intrahepatic recurrence	11 (36.7%)	14 (30.4%)	0.572
Extrahepatic recurrence	3 (10.0%)	4 (8.7%)	0.848

NALP: non-alcoholic patients; ALP: alcoholic patients; fc: formation of capsule; vp: microscopic portal vein invasion; vv: microscopic hepatic vein invasion; im: intrahepatic metastasis; NL: normal liver; CH: chronic hepatitis; LC: liver cirrhosis; PHLF: posthepatectomy liver failure.

**Table 5:** Histopathological examination based on Kleiner's classification.

Item	Score/Code	NALP (*n* = 30)	ALP (*n* = 46)	*P* value
Steatosis	0	24 (80.0%)	39 (84.8%)	0.588
	1	4 (13.3%)	5 (10.9%)	0.745
	2	1 (3.3%)	2 (4.3%)	0.824
	3	1 (3.3%)	0 (0.0%)	0.213
Lobular inflammation	0	1 (3.3%)	0 (0.0%)	0.213
	1	18 (60.0%)	34 (73.9%)	0.202
	2	8 (26.7%)	11 (23.9%)	0.786
	3	3 (10.0%)	1 (2.2%)	0.135
Ballooning	0	20 (66.7%)	35 (77.1%)	0.369
	1	8 (26.7%)	7 (15.2%)	0.220
	2	2 (6.7%)	3 (6.5%)	0.980
Fibrosis	0	2 (6.7%)	0 (0.0%)	0.076
	1	10 (33.3%)	11 (23.9%)	0.369
	2	3 (10.0%)	11 (23.9%)	0.126
	3	6 (20.0%)	11 (23.9%)	0.689
	4	9 (30.0%)	13 (28.3%)	0.870
NAS		2 (0–5)	2 (1–4)	0.290
NAS with fibrosis		4 (0–9)	4 (2–8)	0.996

NALP: nonalcoholic patients; ALP: alcoholic patients; NAS: nonalcoholic fatty liver disease (NAFLD) activity score, NAS of 5 was found in two patients (6.7%).

**Table 6:** Multivariate analysis of factors contributing to overall survival and recurrence-free survival in group NBNC.

Overall survival	Hazard ratio (95% CI)	*P* value
Child-Pugh B/C	19.667 (1.346–287.340)	0.029
im(+)	31.064 (1.965–491.024)	0.015
Extrahepatic recurrence	31.717 (1.261–797.764)	0.036

im: intrahepatic metastasis.
